# Laboratory-confirmed respiratory syncytial virus (RSV) hospitalizations: a national all ages cross-section evaluation, 2020–2024

**DOI:** 10.1186/s13584-025-00693-5

**Published:** 2025-06-11

**Authors:** Aharona Glatman-Freedman, Lea Gur-Arie, Rita Dichtiar, Lital Keinan-Boker, Michal Bromberg, with the Israeli Respiratory Viruses Hospital Laboratory Network (IRVHLN)

**Affiliations:** 1 The Israel Center for Disease Control, Ministry of Health, Ramat Gan, Israel; 2 School of Public Health, Faculty of Medical and Health Sciences, Tel Aviv University, Tel Aviv, Israel; 3 School of Public Health, University of Haifa, Haifa, Israel

## Abstract

**Background:**

New vaccines and monoclonal antibody (mAb) against respiratory syncytial virus (RSV) were recently approved for adults and infants, respectively. However, their inclusion in national vaccination programs has been slow. Accurate assessment of RSV disease burden among all ages is essential for the global introduction of these agents.

**Methods:**

We evaluated all-ages burden of RSV hospitalizations, from 2020 to 2024, based on data collected by a new national laboratory-based hospital surveillance system. RSV-positive respiratory samples from patients hospitalized in general hospitals nationwide were reported. Data were analyzed by RSV circulation periods and age-group to determine hospitalization rates and 30-day mortality (30-DM) rates. We compared the laboratory-confirmed hospitalization rates with rates previously calculated based on ICD-9 codes.

**Results:**

RSV-confirmed hospitalizations were reported for all age-groups. The highest RSV hospitalization rates were found among patients < 1 year old. Patients ≥ 60 years old had the highest RSV hospitalization rates among ≥ 5 years old patients, and their 30-DM rates reached 14.7%, exceeding those of influenza. During the COVID-19 pandemic, lower rates of RSV-confirmed hospitalizations were reported among ≥ 60 years old patients, probably due to higher adherence to social distancing measures. We found higher numbers and rates of laboratory-confirmed hospitalizations among all age-groups ≥ 1 year old, than those previously reported by our group, based on ICD-9 codes.

**Conclusions:**

Laboratory-confirmation of RSV is paramount for optimal assessment of RSV hospitalization burden, particularly beyond infancy, and for the global adoption of newly developed vaccines and mAb.

**Supplementary Information:**

The online version contains supplementary material available at 10.1186/s13584-025-00693-5.

## Introduction

New approaches for the prevention of diseases caused by Respiratory Syncytial Virus (RSV) in infants and adults were recently approved by the Food and Drug Administration (FDA) and by the European Medicines Agency (EMA) [1, 2]. Nirsevimab, a long-acting recombinant human monoclonal antibody (mAb) which targets a highly conserved site of the prefusion conformation of the RSV fusion (F) protein was approved for infants [1, 2]. A single dose of Nirsevimab can provide coverage for the duration of an RSV season in regions that have a clear RSV seasonal pattern [3], due to higher and more sustainable levels of neutralizing antibodies as compared with palivizumab [4]. It was introduced thus far into the public immunization programs of several high-income countries [5]. In Israel it was approved in February 2025 for all infants in their first year of life [6], and is expected to be in use for the 2025–2026 season.

Active RSV vaccines were approved by the FDA and EMA for pregnant women in the third trimester, targeting babies in their first months of life, and for elderly adults [1, 2].

Several high-income countries such as the United Kingdom, Canada and Australia have already included RSV vaccines in their national immunization programs. The United Kingdom included RSV vaccination for adults ≥ 75 years old and pregnant women [7]. Canada included RSV vaccine for ≥ 60 year old adults [8] and Australia included RSV vaccine for pregnant women [9]. In the United States, RSV vaccine is available for older adults who purchase Medicare Part D (Drugs & prescription plan) [10]. However, in other high-income countries, the RSV vaccine costs are carried by the vaccinee.

A recent World Health Organization (WHO) global marketing study, concluded that uncertainty exists regarding the global adoption of RSV vaccines and mAbs, particularly in countries with limited resources [11]. The study suggests that RSV vaccines and mAb became available during challenging times for national immunization programs, characterized by financial difficulties, competition of health priorities and low-uptake for the existing vaccines [11].

Accurate assessment of RSV disease burden is essential for the continued introduction of RSV vaccines and mAbs. Unlike infants, who mostly present with symptoms of bronchiolitis, respiratory diseases caused by RSV in other age-groups, cannot be distinguished from those caused by other respiratory viruses [12]. Although the awareness of RSV morbidity among older adults has increased substantially in recent years, estimation of RSV disease burden beyond infancy, is still incomplete.

A hospital respiratory virus surveillance system was established by the Israel Center for Disease Control (ICDC) in September 2020 [13]. This system, which relies on reports of real-time reverse transcription polymerase chain reaction (RT-PCR) laboratory confirmation of hospitalized patients from all general hospitals in Israel, was found to be both sensitive and reliable [13].

We aimed to evaluate the burden of RSV-confirmed hospitalizations in all age-groups, from 2020 to 2024, based on data of the new surveillance system.

## Methods

### Data

Hospital laboratories reported weekly to the ICDC, RSV-positive respiratory samples from hospitalized patients tested by real-time RT-PCR accompanied by patients’ information as previously described [13]. Reports were communicated via virtual safes for enhanced protection.

When repeated RSV-positive samples were detected within 1 month, for the same patient, only the first RSV-positive result was included.

Data were aggregated by epidemiological week and age-group, and presented as raw numbers, percentages and rates per 100,000 population of the relevant age group.

### RSV circulation periods

The overall evaluation included RSV-positive cases reported between epidemiological week 36, 2020 and week 27 2024, focusing on the periods of active circulation in Israel, each defined as RSV Circulation Period (RCP). The start and the end week of each RCP was determined as the first and last week in which the number of weekly RSV-positive cases was ≥ 10, respectively. Due to unusual circulation patterns of RSV in 2021–2022, the end of the 2021 RCP and the beginning of the 2021–2022 RCP were determined based on the official start of the respiratory viruses’ surveillance period in Israel, which occurs on week 40 each year. 

### Hospitalizations

The number and rate of RSV-positive hospitalizations per 100,000 population were calculated for all hospitalizations and by the age-groups, for each RCPs.

### Mortality

To evaluate the number of deaths among hospitalized RSV-positive cases, patients were examined vis-a-vis the national population registry, to detect deaths occurring within 30 days from sampling date.

30-day mortality (30-DM) rate was calculated as percent deaths of RSV-positive hospitalized patients by age-group. RSV 30-DM rates were compared with those of influenza (for which data were collected and analyzed using the same methodology as for RSV).

### Intensive and enhanced care units

The use of intensive care unit (ICU) or enhanced care units (ECU), was calculated by age-group, based on data from 11 (out of 26) general hospitals. These hospitals provided information about ‘admission department’ and ‘discharge department’. If one of them or both were ICU/ECU, the patient was classified as a patient who used ICU/ECU. Patients who were reported several times within the same hospitalization, and any of those reports included ICU/ECU as the admission or discharge department, were also classified as patients who used ICU/ECU.

### Comparison with previous analysis

To evaluate whether our results were consistent with results of a previous study of our group [14], which relied on International Classification of Diseases 9th Revision (ICD-9) codes, we modified the age-group stratification of the 2020–2024 data, to resemble that of the previous study. Rates of hospitalizations per 100,000 population of the two studies were compared by age-group.

### Statistics

RSV-positive hospitalizations were presented as numbers, percentages and rates. Hospitalizations of each age-group were calculated as percentage of all RSV-positive hospitalizations for the specific RCP as well as for all RCPs together. Rates per 100,000 population for each age-group were calculated using the age-specific population size obtained from the Israel Central Bureau of statistics (CBS) [15]. ICU/ECU hospitalizations were expressed as percentage. Differences in rates were evaluates by *χ*^2^ test or Mantel–Haenszel test. *p* value (2-tailed) of < 0.05 was considered statistically significant. Statistical analysis was performed using Open Epi statistical software Version 3.01 (www.OpenEpi.com).

### Ethical considerations

This evaluation was reviewed by the National Helsinki Committee for Human Medical Research of the Israel Ministry of Health; it was determined to be part of the Ministry of Health’s professional activity, which is presented in an anonymized and aggregated form, and as such, does not require an ethical committee’s approval or informed consent.

## Results

### Population and RSV circulation periods

From week 36 to week 53, 2020, which occurred during active SARS-CoV-2 circulation, only 3 RSV cases were identified. From week 1 2021 to week 27 2024 a total of 19,799 RSV-positive hospitalizations were reported. Age was verified for 17,812 (90%) of them.

**Fig. 1:**
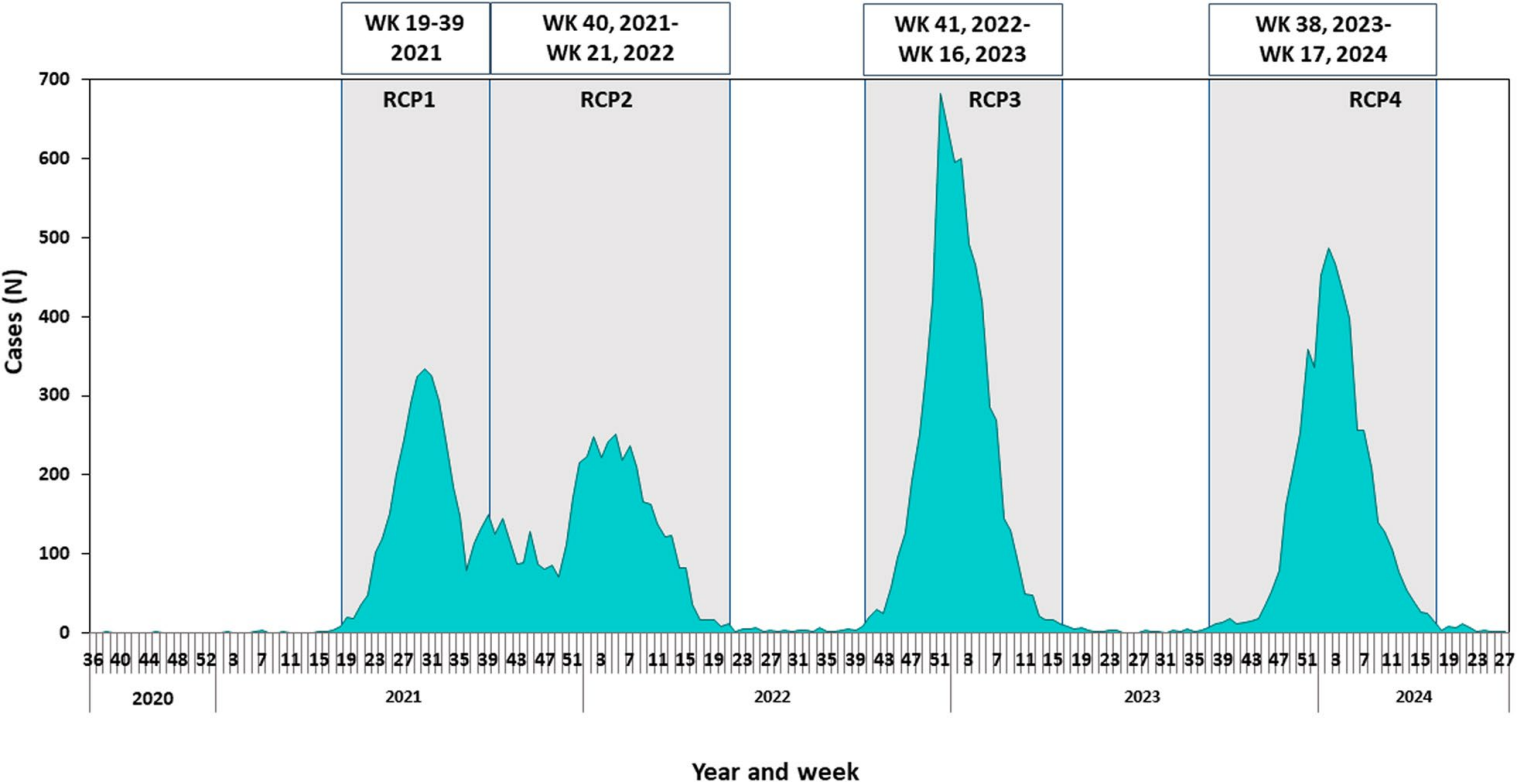
Weekly RSV-confirmed hospitalizations in general hospitals 2020–2024, highlighting RSV circulation periods (marked by grey areas)

Four RCPs occurred during the evaluation period. The first RCP occurred during the spring–summer of 2021 (Fig. 1). The remaining three RCPs occurred during the fall-winter seasons of 2021–2022, 2022–2023 and 2023–2024. The periods of RSV circulation are detailed in Fig. 1 and in Table 1S.

### Hospitalizations

**Fig. 2:**
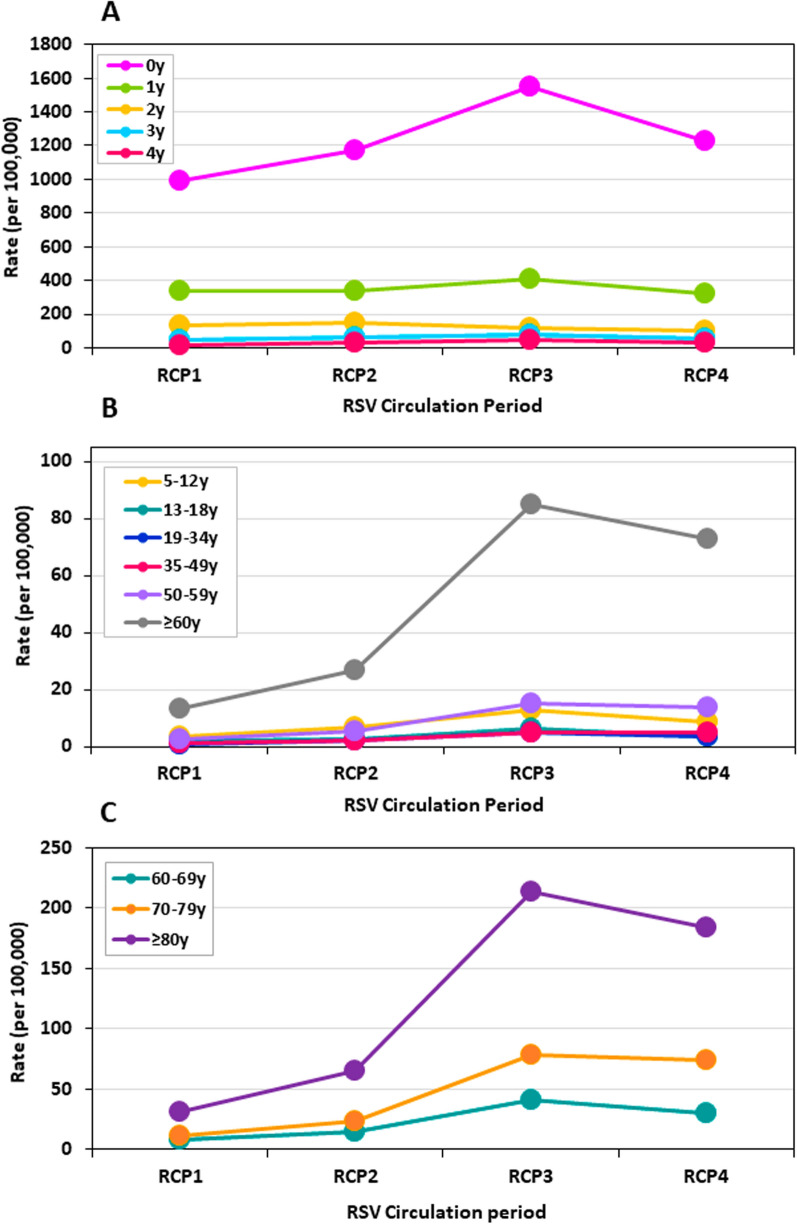
Rates of RSV-positive hospitalizations per 100,000 population, by age-group, and by RSV circulation period. **A** Infants and children < 5 years of age. **B** ≥ 5 years of age. **C** ≥ 60 years of age

Of the total of 17, 812 hospitalizations, with verified patients’ age, reported between week 1 2021 and week 27 2024, 17, 629 (99.0%) hospitalizations occurred during the four RCPs. Table 1 demonstrates the hospitalizations frequencies, percentages and rates for each RCP, by age group. Age-group rates per 100,000 population are shown in Fig. 2.

**Table 1:** Numbers, percentages and rates of RSV-confirmed hospitalizations by RSV Circulation Periods (RCPs) and age-groups

RCP	RCP1			RCP2			RCP3			RCP4			All RCPs		
	(N)	(%)	Rate/10^5^	(N)	(%)	Rate/10^5^	(N)	(%)	Rate/10^5^	(N)	(%)	Rate/10^5^	Total (N)	Total (%)	Mean rate/10^5^
*Age (years)*															
0	1803	58.0	987.9	2145	55.3	1175.3	2829	47.8	1550.1	2,235	47.3	1224.7	9012	51.1	1234.5
1	609	19.6	334.8	613	15.8	337.0	746	12.6	410.1	582	12.3	320.0	2550	14.5	350.5
2	234	7.5	129.5	267	6.9	147.8	212	3.6	117.3	186	3.9	102.9	899	5.1	124.4
3	93	3.0	50.3	117	3.0	63.2	145	2.5	78.4	94	2.0	50.8	449	2.5	60.7
4	26	0.8	13.9	64	1.7	34.3	87	1.5	46.6	57	1.2	30.5	234	1.3	31.4
5–12	53	1.7	3.7	95	2.4	6.7	180	3.0	12.7	120	2.5	8.4	448	2.5	31.5
13–18	19	0.6	2.0	25	0.6	2.7	59	1.0	6.3	32	0.7	3.4	135	0.8	14.5
19–34	17	0.5	0.8	44	1.1	2.1	105	1.8	5.0	74	1.6	3.5	240	1.4	0.0
35–49	20	0.6	1.2	39	1.0	2.3	85	1.4	4.9	85	1.8	4.9	229	1.3	13.2
50–59	23	0.7	2.6	47	1.2	5.3	136	2.3	15.3	121	2.6	13.6	327	1.9	43.6
≥ 60	209	6.7	13.3	422	10.9	26.9	1334	22.5	85.0	1141	24.1	72.7	3106	17.6	197.9
Total	3106	100.0	32.5	3878	100.0	40.6	5918	100.0	61.9	4727	100.0	49.5	17,629	100.0	184.5
60–69	58	1.9	7.7	108	2.8	14.4	309	5.2	41.2	228	4.8	30.4	703	4.0	93.7
70–79	64	2.1	11.9	130	3.4	24.1	426	7.2	79.0	398	8.4	73.8	1018	5.8	188.8
≥ 80	87	2.8	31.0	184	4.7	65.6	599	10.1	213.5	515	10.9	183.5	1385	7.9	493.6

Infants < 1 year old (0 year) had the highest proportions of RSV-positive hospitalizations in all four RCPs, ranging from 47.3% (RCP4) to 58.0% (RCP1) (Table 1).

Adults ≥ 60 years of age had the second highest proportions of RSV-positive hospitalizations in RCP3 and RCP4, constituting 22.5% and 24.1%, respectively. However, in RCP1, this age-group had the fourth, and in RCP2 it had the third highest proportion of RSV-positive hospitalizations, constituting 6.7% and 10.9%, respectively (Table 1).

Since a total of 4,485 RSV-positive hospitalizations were reported among patients ≥ 5 years old in the 4 RCPs, the 3,106 RSV-positive hospitalizations reported for patients ≥ 60 years old in all 4 RCPs, established that this age-group had the highest proportion (69.3%) of hospitalizations among patients ≥ 5 years old (Table 1).

Two age-groups demonstrated substantial rate differences among the RCP periods: infants < 1 year and adults ≥ 60 years old.

Specifically, hospitalization rates among infants < 1 year old were highest in RCP3, and lowest in RCP1 as compared with the other RCPs (Table 1, Fig. 2A). The differences in rates among the RCPs in < 1 year old patients were statistically significant (*p* value < 0.01). Hospitalization rates among ≥ 60 years old patients were substantially higher in RCP3 and RCP4 as compared with RCP1 and RCP2. The largest difference, was observed between RCP2 and RCP3 (3.2-fold difference) (Table 1, Fig. 2B). The rate differences among the RCPs in ≥ 60 years old patients were statistically significant (*p* value < 0.01).

### Hospitalizations among infants < 1 year old

**Fig. 3:**
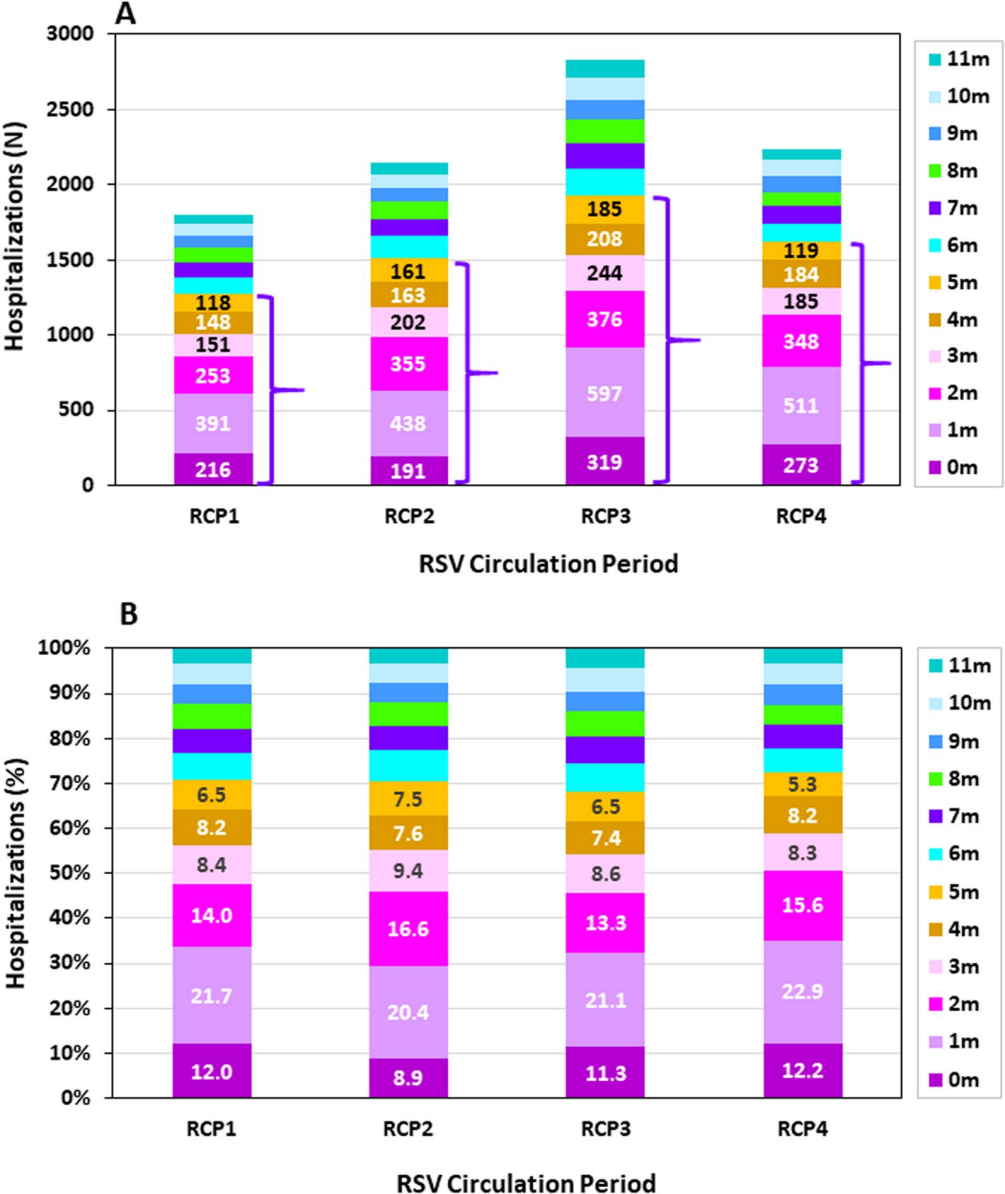
RSV-confirmed hospitalizations among infants in their first year of life, by month of life and RSV circulation period (RCP). **A** Numbers, **B** Percentages

Figure 3 demonstrates the distribution of RSV-positive hospitalizations by month of life among infants < 1 year old in the four RCPs.

The highest percentage of RSV-positive infants were in the second month of life (1 m) averaging 21.5% (SD 1.0), followed by infants in their third month of life (2 m) averaging 14.9% (SD 1.5), and infants in their first month of life (0 m) averaging 11.1% (SD 1.5) (Fig. 3B).

Infants less than 3 month of age constituted, on average, 47.5% (SD 2.3) of RSV-positive hospitalized < 1 year old infants, ranging from 50.6% during RCP4 to 45.7% during RCP3 (Fig. 3B).

Infants less than 6 month of age constituted, on average, 70.5% (SD 1.8) of < 1 year old infants, ranging from 72.5% (SD 1.8) during RCP4 to 68.2% during RCP3 (Fig. 3B).

### *Hospitalizations among adults* ≥ *60 years old*

Table 1 and Fig. 2C demonstrate the numbers and rates of RSV-positive cases ≥ 60 years by age subgroups. Among RSV-positive cases of the ≥ 60 years, the ≥ 80 years old subgroup was the largest (Table 1, Fig. 2C). The ≥ 80 years old subgroup demonstrated the largest increase in the number and rates of cases between RCP2 and RCP3. While during RCP3 and RCP4, the ≥ 80 years subgroup was the third largest group, after the 0 and 1 year old age-groups, during RCP1 and RCP2, this subgroup was the fifth and fourth largest group, respectively (Table 1, Fig. 2C). For all three age subgroups of the ≥ 60 years old age-group, the hospitalizations rate differences among the four RCPs were statistically significant (*p* value < 0.01).

### Mortality

Table 2 shows the 30-DM for all age-groups and age subgroups. Patients ≥ 80 years, had the highest 30-DM in all RCPs, ranging between 15.73% and 19.20%, followed by the 70–79 years age subgroup, which ranged between 9.38% and 13.85%. Patients 60–69 years had the 3rd highest 30-DM during RCP3 and RCP4, the 4th highest 30-DM during RCP2, and the 5th during RCP1 (Table 2). Patients in their first and second year of life had 30-DMs of ≤ 0.11% and ≤ 0.33%, respectively, in all RCPs (Table 2).

**Table 2:** 30-day mortality (30-DM) among RSV-positive hospitalized cases, by RSV circulation periods and age group

Age (years)	RCP1			RCP2			RCP3			RCP4		
	RSV-positive (N)	Deaths by 30 days (N)	30-DM (%)	RSV-positive (N)	Deaths by 30 days (N)	30-DM (%)	RSV-positive (N)	Deaths by 30 days (N)	30-DM (%)	RSV-positive (N)	Deaths by 30 days (N)	30-DM (%)
0	1803	2	0.11	2145	1	0.05	2829	1	0.04	2235	0	0.00
1	609	2	0.33	613	0	0.00	746	2	0.27	582	1	0.17
2–4	353	1	0.28	448	0	0.00	444	0	0.00	337	1	0.30
5–12	53	1	1.89	95	0	0.00	180	0	0.00	120	1	0.83
13–18	19	0	0.00	25	0	0.00	59	2	3.39	32	0	0.00
19–35	17	0	0.00	44	2	4.55	105	1	0.95	74	1	1.35
35–50	20	1	5.00	39	2	5.13	85	1	1.18	85	4	4.71
50–59	23	1	4.35	47	4	8.51	136	11	8.09	121	4	3.31
≥ 60	209	22	10.53	422	62	14.69	1334	189	14.17	1141	154	13.50
Total	3106	30	1.0	3878	71	1.83	5918	207	3.49	4727	166	3.51
60–69	58	2	3.45	108	9	8.33	309	25	8.09	228	21	9.21
70–79	64	6	9.38	130	18	13.85	426	49	11.50	398	52	13.07
≥ 80	87	14	16.09	184	35	19.02	599	115	19.20	515	81	15.73

**Fig. 4:**
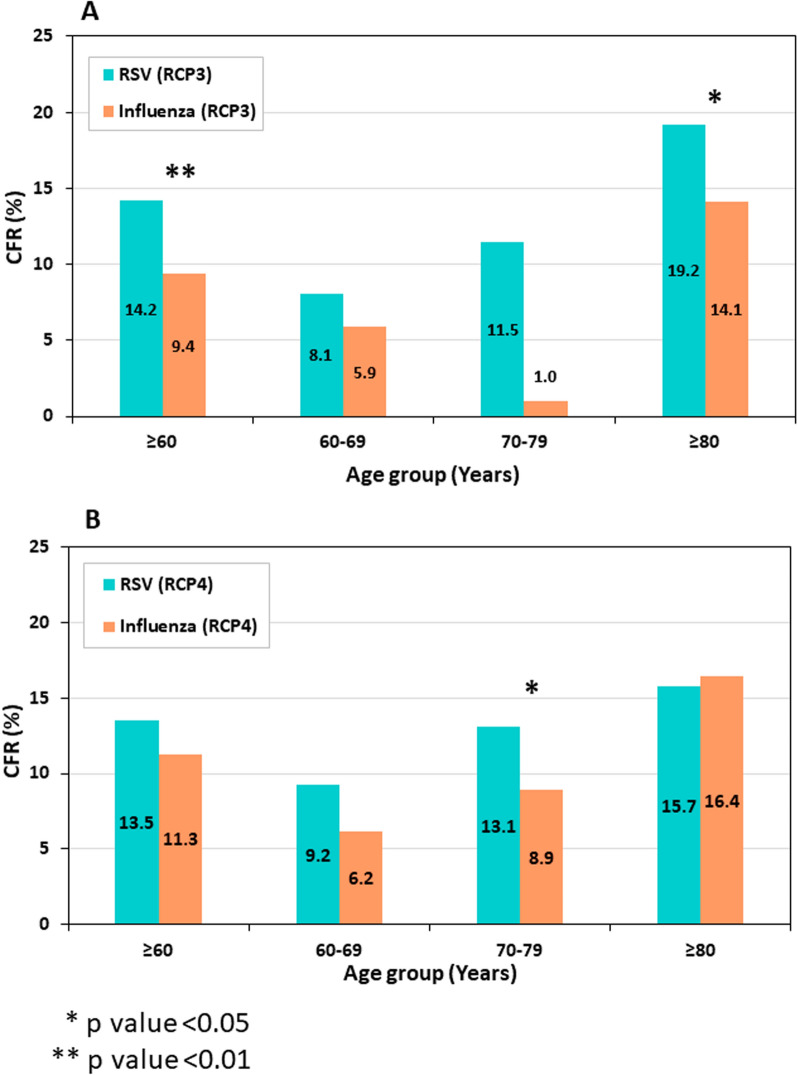
30-day mortality (30-DM) rates for RSV-confirmed (green bars) and Influenza-confirmed (orange bars) hospitalizations among individuals ≥ 60 years of age. **A** RCP3. **B** RCP4

Comparison between the 30-DM of RSV-positive and that of influenza-positive patients ≥ 60 years old, was performed for the two post COVID-19 RCPs (namely RCP-3 and RCP-4), in which both viruses’ activity returned to their pre-pandemic patterns. 30-DM was higher for RSV-positive compared with influenza-positive hospitalized patients (Fig. 4A, B). The differences were statistically significant for the entire ≥ 60 years old age-group in RCP3 (*p* value < 0.01), but not in RCP4 (*p* value 0.052). Age subgroup analysis demonstrated RSV 30-DM was significantly higher than influenza 30-DM in the ≥ 80 years old subgroup in RCP3 (*p* value < 0.05) (Fig. 4A) and for the 70–79 years old subgroup in RCP4 (*p* value < 0.05) (Fig. 4B).

### Intensive and enhanced care units

Evaluation of the use of ICU/ECU was based on data from 11 (out of the 26) general hospitals in Israel for RCP3 and ECP4 in which both viruses’ activity returned to their pre-pandemic patterns. A total of 152 RSV-positive hospitalized patients in RCP3, and 91 in RCP4, required ICU/ECU, constituting 5% and 3.7% of the total hospitalizations, respectively. The highest numbers of ICU/ECU hospitalizations occurred among infants < 1 year old and ≥ 60 years old patients in both RCPs, constituting together 82.2% and 74.7% of ICU/ECU patients in RCP3 and RCP4, respectively (Table 3). ICU/ECU patients of all the other age-groups together, constituted 17.8% and 25.3% of ICU/ECU patients in RCP3 and RCP4, respectively (Table 3).

**Table 3:** Numbers and rates of intensive care unit/enhanced care unit utilization among RSV-positive hospitalized patients, RCP3 and RCP4

Age (years)	RCP3				RCP4			
	ICU/ECU (N)	No ICU/ECU (N)	Total (N)	ICU/ECU (%)	ICU/ECU (N)	No ICU/ECU (N)	Total (N)	ICU/ECU (%)
0	78	1341	1419	5.5	42	1075	1117	3.8
1	11	365	376	2.9	6	321	327	1.8
2–4	2	231	233	0.9	7	204	211	3.3
5–12	5	86	91	5.5	1	65	66	1.5
13–18	0	23	23	0.0	1	11	12	8.3
19–35	5	39	44	11.4	3	42	45	6.7
35–50	2	40	42	4.8	2	47	49	4.1
50–59	2	67	69	2.9	3	57	60	5.0
≥ 60	47	702	749	6.3	26	528	554	4.7
Total	152	2894	3046	5.0	91	2350	2441	3.7
60–69	11	153	164	6.7	12	119	131	9.2
70–79	12	221	233	5.2	9	180	189	4.8
≥ 80	24	328	352	6.8	5	229	234	2.1

### Comparison of RSV hospitalizations analyses

Table 2S demonstrates the rates per 100,000 of 2021–2024 laboratory-confirmed RSV hospitalizations alongside those of RSV-related primary hospital discharge ICD.9 diagnoses from our 2000–2017 study [14].

The rates for infants < 1 year of age were similar in the two analyses. However, the mean rates of all other age groups were higher in the 2021–2024 laboratory-based evaluation, as compared to those of the 2000–2017 RSV-related primary ICD.9-based analysis. The differences were more pronounced among individuals ≥ 3 years old (Table 2S).

Evaluation of the 2021–2024 rates vis-à-vis the 2017 rates, a year for which > 90% of the primary ICD.9 diagnoses were RSV-specific, demonstrated similar trends, with higher 2021–2024 rates for individuals ≥ 1 years old, and with larger differences among hospitalized patients ≥ 3 years old (Table 2S).

## Discussion

In all four RCPs evaluated, the highest numbers and rates of RSV-confirmed hospitalizations was observed among infants < 1 year old, with the ≥ 60 years old age-group having the highest numbers and rates among ≥ 5 years old patients.

The RCP1 and RCP2, differed from RCP3 and RCP4 in seasonal patterns and in the rate of hospitalizations among age-groups. The differences were particularly noticeable among ≥ 60 years old patients, and to a lesser extent among 50–59 years old and 35–49 years old patients, with rates being substantially higher during RCP3 and RCP4. These differences may be due to the SARS-CoV-2 pandemic, which occurred in waves during the years 2020–2022 and affected the circulation of other respiratory viruses [13, 16]. Specifically, RCP1 and RCP2 coincided with the Delta and the first Omicron wave in Israel, respectively [13].

Although no official lockdown was declared in Israel during the Delta and Omicron circulation, other social distancing measures were implemented [17, 18], creating less opportunity for RSV transmission between children and elderly adults.

It is important to note that based on an ICDC syndromic surveillance platform, which monitors emergency department visits due to bronchiolitis among infants less than 2 years of age, the timing and seasonal pattern of the 2022–2023 and 2023–2024 were similar to those of the pre-pandemic RCPs [19]. Thus, it is highly likely that RCP3 and RCP4 were similar to pre-pandemic RCPs also with regard to other characteristics such as age group distribution and the use of ICU/ECU.

We demonstrated a substantial use of ICU/ECU care among ≥ 60 years old patients, constituting the second largest age-group among ICU/ECU patients, after the < 1 year old patients. Furthermore, patients ≥ 60 years of age also had the highest 30-DM rate of all age-groups.

Stratification of RSV hospitalizations among ≥ 60 years old patients, demonstrated that the highest hospitalizations numbers, hospitalization rates and 30-DM rates, in all four RCPs, were found among ≥ 80 years old patients. These data are important for prioritizing RSV vaccines among ≥ 60 years old individuals, particularly in limited-resources circumstances.

The similar hospitalization rates in the first year of life, in both Laboratory-based and ICD-9-based evaluations, may be due to the clear clinical presentation of bronchiolitis, which is highly suggestive of RSV, and to the fact that RSV laboratory confirmation tests have been regularly performed for patients of this age-group, in Israel, for many years.

The higher rates of RSV-confirmed hospitalizations among patients ≥ 1 year old in the current evaluation, compared with our previous ICD-9 based evaluation, is probably due to the substantially higher usage of RSV PCR testing, among all age-groups in recent years, as well as medical teams’ awareness of RSV morbidity beyond infancy or early childhood. Furthermore, in the absence of a unique clinical manifestation of RSV beyond infancy, the usage of RSV ICD-9 codes, among these age-groups, may be sub-optimal. ICD coding omissions were previously shown in a variety of medical conditions [20–22]. Some of these omissions resulted from viral laboratory results becoming available only after hospital discharge [23] or from misclassification. In this regard, a recent study from Denmark demonstrated that more than 50% of RSV-positive hospitalized adults did not receive an RSV-specific ICD-10 code [24].

Determination of RSV as cause of death can be challenging. Inaccuracies in determining cause of death were reported in multiple studies [25–31] for multiple diagnoses. In the case of RSV, the issues related to the use of ICD codes, discussed above, probably apply also for determination of cause of death. For this reason, in this study, we assessed mortality among RSV-positive hospitalized patients, by evaluating deaths occurring withing a specified time following sampling, without using death certificates. This assessment, which is based on two documented events (sampling and death date within 30 days), allowed side-stepping the contribution of variability due to human misclassification.

We found that 30-DM rates among RSV-positive ≥ 60 years old patients, were higher than those of influenza-positive patients of the same age group. In this regard, a recent study from Japan, covering 13 years and 56,980 patients, demonstrated that in-hospital mortality among ≥ 60 years old RSV patients was significantly higher than that of ≥ 60 years old influenza patients [32].

Many studies on RSV hospitalizations, have used ICD-9/ICD-10 diagnoses codes and administrative data [14, 33–36], while others used a variety of laboratory methods [35, 37]. Only a few studies on hospitalization burden used PCR laboratory confirmation exclusively. However, most evaluated either children [38–41] or adults [42–44]. Although one laboratory-based study, evaluated all ages, it addressed only patients fulfilling the case definition of Severe Acute Respiratory Infection (SARI), which requires the presence of both fever and cough [45]. Thus, the use of SARI case definition is likely to miss RSV patients who present with other symptoms, or present with either cough or fever.

The principal advantages of our evaluation, is the use of national-level data, inclusion of all ages, and reliance only on a single, sensitive method (PCR) for RSV laboratory-confirmation. The combination of these advantages, make our evaluation the first of this kind, to the best of our knowledge. Our evaluation also allowed the demonstration of changes in RSV age-group distribution during the COVID-19 pandemic.

Our study has several limitations. Although, we were able to confirm the presence of RSV among hospitalized patients, the exact reason for hospital admission, PCR testing or death were unavailable. Furthermore, some variations in PCR laboratory processes, may exist among laboratories. Although, these limitations could potentially lead to some misclassification, given the high volume of respiratory virus PCR testing performed, by all the reporting medical centers, the cumulative results are likely to reflect both viral circulation and level of disease activity. Furthermore, the proportions of RSV-confirmed hospitalizations burden and 30-DM rates among different age-groups, are less likely to be affected.

Our evaluation did not include medical data of the RSV-positive hospitalized patients. Therefore, we could not assess risk factors such as comorbidities or demographic characteristics, the course of disease or the use of certain medical resources such as mechanical ventilation. While assessment of risk factors and the use of medical resources are of great importance, they require a separate evaluation, one which relies on medical records’ data and a different study design.

Although the use of 30-day mortality provides a unified method for comparing short-term mortality among hospitalized patients, long-term outcomes are also important. In this regard, a recent study from Japan, covering 13 years and 56,980 adult patients, demonstrated that ≥ 60 years old patients with RSV had a higher risk of readmission within 1 year due to respiratory diseases or any other cause, and a higher 1-year mortality as compared with influenza patients [32].

Our evaluation did not include assessment of the burden of community-level RSV-related illness. However, since the patterns of RSV testing and outpatient clinic visits differ from those of hospitalizations, such assessment requires the use of different data sources and a different study design.

In conclusion, our study provides a national view on the distribution and impact of RSV-documented hospitalizations across all ages, providing evidence for the relative weight of hospitalizations, specifically among age-groups that are targeted for passive or active vaccination.

### Implications for public policy

The data presented here were made available to the 2025 Israel Public Committee for the Expansion of Healthcare Services Basket. Both passive and active vaccines against RSV were submitted to the 2025 committee, for use in infants, pregnant women and adults ≥ 60 years old [46]. In February 2025, the committee approved the use of NirsevimAb for infants in their first year of life, in Israel [6]. Our data show that RSV-confirmed hospitalizations among infants in their first year of life constitute about half of all RSV-confirmed hospitalizations, making it the age-group with the highest number of hospitalizations. Thus, the approval of NirsevimAb by the Healthcare Services Basket committee, for this age-group, is merited and well-supported by the data presented here. It is yet to be seen whether vaccinating infants with NirsevimAb will affect the burden of hospitalizations among other age groups. However, the topic has already been raised by several groups [47–49], and two modelling studies suggested that RSV-related medically attended LRTI and hospitalizations in the general population can be averted as well [47, 49], especially when infant NirsevimAb coverage is high. In this regard, a recent household transmission study from the United States found that the direction of most household RSV transmission was from infants and young children to adults [50]. Social support in Israel ranks among the highest in the world [51], and among its common expressions are close contacts among infants, children and elderly adults, including grandparents. This intergenerational contact consists of, among others, extended family gatherings on weekends and holidays, or grandparents caring for young infants and children during the latter's acute illness, allowing parents to attend the workplace. Thus, passive vaccination of < 1 year old infants with long-acting mAb, may lower infants’ disease rates as well as RSV transmission to other family members, providing indirect protection.

Early studies demonstrated NirsevimAb effectiveness among infants of 81% to 83% against RSV-confirmed hospitalizations [52–55], and 87% to 91% against RSV-confirmed hospitalizations requiring supplemental oxygen [52, 54]. These results further support the policy decision made by the Healthcare Services Basket committee. Evaluations of NirsevimAb uptake, effectiveness and impact in Israel, are warranted, and should provide insight into the magnitude of both its direct and indirect protection. Thus, continuing laboratory-confirmed RSV hospitalizations surveillance is paramount for evaluating on-going disease burden, in the presence of new vaccines.

Recent studies among ≥ 60 years old adults demonstrated an active RSV vaccine effectiveness (VE) of 91% (95% CI 59–98) against RSV-related lower respiratory tract disease (LRTD) emergency department visits and hospitalizations [56], VE of 80% (95% CI 71–85) against RSV-related hospitalizations, and VE of 81% (52–92) against RSV-related severe illness, among non-immunocompromised patients [57]. A large international multicenter study showed that RSV vaccine, administered to pregnant women at least 14 days before delivery, had an efficacy of 81.8% (99.5% CI 40.6–96.3) against severe LRTD among infants within 90 days from birth, and 69.4% (97.58% CI 44.3–84.1) within 180 days from birth [58].

The results of our evaluation will be made available also to future Healthcare Services Basket Committees, which are expected to discuss the addition of active RSV vaccines for elderly adults and pregnant women.

## Supplementary Information

Additional file 1.

Additional file 2.

## Data Availability

Data is not publicly available. Requests for data can be addressed to: Aharona Glatman-Freedman, Israel Center for Disease control, Ministry of Health, Israel Email: aharona.freedman@moh.gov.il.
