# Supplementary material for: Laboratory-confirmed respiratory syncytial virus (RSV) hospitalizations: a national all ages cross-section evaluation, 2020–2024
Source: Isr J Health Policy Res. 2025 Jun 11;14:36. doi: 10.1186/s13584-025-00693-5 (PMC12153084; doi:10.1186/s13584-025-00693-5)
Supplement: Supplementary file 1 — Additional file 1. [file 13584_2025_693_MOESM1_ESM.docx]

| **Season/off-season** | **End week,year** | **Start week, year** | **Year** | **RSV**  **circulation period (RCP)** |
| --- | --- | --- | --- | --- |
| Off-season | Week 39, 2021 | Week 19, 2021 | 2021 | 1 |
| In-season | Week 21, 2022 | Week 40, 2021 | 2021-2022 | 2 |
| In-season | Week 16, 2023 | Week 41, 2022 | 2022-2023 | 3 |
| In-season | Week 17, 2024 | Week 38, 2023 | 2023-2024 | 4 |

Table 1S. Periods of enhanced RSV activity within the evaluation period
