# Supplementary material for: Laboratory-confirmed respiratory syncytial virus (RSV) hospitalizations: a national all ages cross-section evaluation, 2020–2024
Source: Isr J Health Policy Res. 2025 Jun 11;14:36. doi: 10.1186/s13584-025-00693-5 (PMC12153084; doi:10.1186/s13584-025-00693-5)
Supplement: Supplementary file 2 — Additional file 2. [file 13584_2025_693_MOESM2_ESM.docx]

| **Rate Ratio** | **Rate Ratio** | **Laboratory-confirmed RSV**  **Rates/10^5^** | **Primary RSV-related ICD.9 diagnoses**  **Rates/10^5^** | |  |
| --- | --- | --- | --- | --- | --- |
| **2021-2024 / 2017** | **2021-2024 / 2000-2017** | **2021-2024**  **Mean rate/10^5^** | **2017** | **2000-2017**  **Mean rate/10^5^** | **Age (years)** |
| 0.97 | 1.01 | 1234.5 | 1269.3 | 1218.4 | 0 |
| 2.15 | 3.01 | 350.5 | 163.3 | 116.4 | 1 |
| 2.99 | 5.65 | 124.4 | 41.6 | 22.0 | 2 |
| 9.79 | 9.95 | 60.7 | 6.2 | 6.1 | 3 |
| 4.98 | 11.21 | 31.4 | 6.3 | 2.8 | 4 |
| 6.71 | 13.42 | 9.4 | 1.4 | 0.7 | 5-9 |
| 11.75 | 23.50 | 4.7 | 0.4 | 0.2 | 10-14 |
| 14.00 | 14.00 | 2.8 | 0.2 | 0.2 | 15-24 |
| 10.00 | 15.00 | 3.0 | 0.3 | 0.2 | 25-44 |
| 8.91 | 2.75 | 9.8 | 1.1 | 0.4 | 45-64 |
| 6.52 | 25.78 | 59.3 | 9.1 | 2.3 | ≥65 |

**Table 2S. Rates per 100,000 population of laboratory-confirmed RSV 2021-2024 vs primary ICD.9 RSV-related diagnosis codes 2000-2017, by age group**
